# NeuroSim Simulator for Compute-in-Memory Hardware Accelerator: Validation and Benchmark

**DOI:** 10.3389/frai.2021.659060

**Published:** 2021-06-09

**Authors:** Anni Lu, Xiaochen Peng, Wantong Li, Hongwu Jiang, Shimeng Yu

**Affiliations:** School of Electrical and Computer Engineering, Georgia Institute of Technology, Atlanta, GA, United States

**Keywords:** compute-in-memory, hardware accelerator, deep neural network, design automation, benchmarking and validation

## Abstract

Compute-in-memory (CIM) is an attractive solution to process the extensive workloads of multiply-and-accumulate (MAC) operations in deep neural network (DNN) hardware accelerators. A simulator with options of various mainstream and emerging memory technologies, architectures, and networks can be a great convenience for fast early-stage design space exploration of CIM hardware accelerators. DNN+NeuroSim is an integrated benchmark framework supporting flexible and hierarchical CIM array design options from a device level, to a circuit level and up to an algorithm level. In this study, we validate and calibrate the prediction of NeuroSim against a 40-nm RRAM-based CIM macro post-layout simulations. First, the parameters of a memory device and CMOS transistor are extracted from the foundry’s process design kit (PDK) and employed in the NeuroSim settings; the peripheral modules and operating dataflow are also configured to be the same as the actual chip implementation. Next, the area, critical path, and energy consumption values from the SPICE simulations at the module level are compared with those from NeuroSim. Some adjustment factors are introduced to account for transistor sizing and wiring area in the layout, gate switching activity, post-layout performance drop, etc. We show that the prediction from NeuroSim is precise with chip-level error under 1% after the calibration. Finally, the system-level performance benchmark is conducted with various device technologies and compared with the results before the validation. The general conclusions stay the same after the validation, but the performance degrades slightly due to the post-layout calibration.

## Introduction

State-of-the-art deep neural network (DNN)–based machine learning algorithms have demonstrated remarkable effectiveness for various artificial intelligence applications such as image processing, speech recognition, and language translation (Deng et al., 2020). However, due to the requirement of high parallelism and power consumption for data movement, computing platforms with traditional von Neumann architecture are inadequate for efficient processing of DNNs. Compute-in-memory (CIM) is a promising solution to alleviate the memory access bottleneck and has achieved attractive energy efficiency when implemented with mature SRAM technology at 7 nm (Dong et al., 2020). With recent progress in emerging nonvolatile memory (eNVM) devices such as resistive random access memory (RRAM) (Xue et al., 2020), phase change memory (PCM) (Burr et al., 2015), and ferroelectric field-effect transistor (FeFET) (Dutta et al., 2020), the application of a CIM-based DNN accelerator is even more intriguing since eNVMs offer low leakage power and nonvolatility which are necessary for dynamic power gating and instant on and off operations in smart edge devices.

However, the performance of CIM can be highly dependent on design factors such as sub-array size, analog-to-digital converter (ADC) precision, and device conductance. Though accurate, the circuit-level SPICE simulation requires dramatically increasing time with the scale of the DNN model. Therefore, a design automation simulator that supports fast modeling of CIM accelerators with various memory technologies and flexible architecture topologies is required to realize an early-stage design space exploration. Among all the reported CIM simulators, NeuroSim (Chen et al., 2018) stands out as a comprehensive platform as it covers a wide variety of design options from a device level to a circuit level and up to an algorithm level. The inputs to the simulator include memory types, nonideal device parameters, transistor technology nodes, network topology and sub-array size, and training dataset and traces. The outputs of the simulator include the hardware performance metrics, such as area, latency, dynamic energy and leakage power consumption, and algorithm-level training/inference accuracy in the run-time. NeuroSim is interfaced with PyTorch, forming an end-to-end benchmark framework, namely, DNN+NeuroSim (Peng et al., 2019), which is publicly available at GitHub with hundreds of users including industry researchers from Intel, Samsung, TSMC, and SK Hynix.

To our best knowledge, *none of other CIM simulators have been validated with the actual silicon data*, although the peripheral circuit modules (e.g., decoder, switch matrix, mux, and adder) of NeuroSim have been validated with SPICE simulations using the PTM model (PTM, 2011) and FreePDK (FreePDK, 2014). It is known that the PTM model and FreePDK are for educational purposes, rather than for foundry fabrication purposes. Therefore, it is imperative to validate the simulator’s prediction with the silicon implementation. In this study, we will validate NeuroSim against a 40-nm 16-kb CIM macro using the TSMC 40-nm RRAM process (Chou et al., 2018), which has been taped out recently (Li et al., 2021). First, the parameters of the memory device and CMOS transistor are exacted from the TSMC’s PDK and employed in the NeuroSim settings. Next, the comparison is made on the analog and digital modules, respectively. New modules such as a level shifter, which uses I/O transistors (to support RRAM’s high write voltage), is added to NeuroSim libraries. The area, critical path delay, and energy consumption are evaluated between the analytical modeling and the SPICE simulations from Cadence Spectre. Finally, adjustment factors are introduced to tune the transistor size, add the wiring area in layout, consider the gate switching rate and the post-layout performance drop, etc. Using the validated NeuroSim settings, we will further benchmark CIM accelerators with a variety of device technologies and compare the performance prediction before and after the validation. It is noted that we only focus on the hardware performance validation in this work and do not focus on the software accuracy estimation, though the inference accuracy is reportable from the framework.

## Background

**FIGURE 1:**
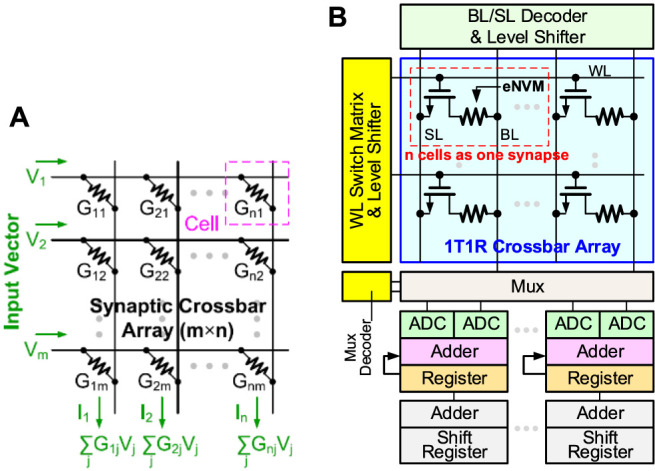
**(A)** Weighted sum (MAC) operation in a conceptual crossbar array structure. **(B)** RRAM-based sub-array with peripheral circuits (e.g., CIM macro).

The convolution neural network (CNN) is one of the most popular DNN models, consisting of multiple convolutional layers to learn the salient features and a few fully connected layers for classification. In this study, we focus on the acceleration of the inference engine where the weights have been pretrained offline. In a convolutional layer, an output feature map (OFM) is the result of multiply-and-accumulate (MAC) operations on a collection of weights (or filters) operating in a sliding window fashion over the input feature map (IFM). Consider the case where the IFM of size W×W×D is processed by N filters, each of size K×K×D. Then the OFM of size W×W×N is computed as follows:

$$O\left[x\right]\left[y\right]\left[n\right]=\;\overset{K-1}{\underset{i=0}{\sum }}\overset{K-1}{\underset{j=0}{\sum }}\overset{D-1}{\underset{k=0}{\sum }}I\left[x+i\right]\left[y+j\right]\left[k\right]\times W\left[i\right]\left[j\right]\left[k\right]\left[n\right],$$

where I, W, and O are the IFM, weights, and OFM, respectively. CIM is an attractive solution for the extensive MAC operations in DNN inference as it combines memory access and computation. The conceptual crossbar structure for CIM is shown in Figure 1A, where the memory device is located at each cross point. If the weights are programmed as the conductance of the memory devices, when the input vectors encoded by read voltage signal, the weighted sum (MAC) operation can be performed in a parallel fashion and obtained as currents at the end of each column. Resistive random access memory (RRAM) is a two-terminal nonvolatile memory based on the metal/oxide/metal structure that stores the multi-bit weight by changing cell’s multilevel conductance states. RRAM has been successfully demonstrated in industrial 40 nm (Chou et al., 2018) and 22 nm platform (Xue et al., 2020). The one-transistor-one-resistor (1T1R) structure is widely used in RRAM-based CIM macro where the word-line (WL) to switch rows of cells and the MAC results are read out through bit-line (BL) voltage converted from weighted sum currents. As shown in Figure 1B, a complete RRAM-based CIM macro also contains peripheral circuits such as a WL switch matrix and BL/SL decoder (to select specific rows or columns), level shifter (to convert the logic V_DD_ to high write voltage for RRAM), MUX and its decoder, analog-to-digital converter (ADC), shift-add, and accumulator to support multi-bit input and multi-bit weight operations.

## NeuroSim Settings

NeuroSim is designed for the CIM-based hardware accelerators. The hierarchy of the simulator consists of different levels of abstraction and analytical modeling from the memory cell and transistor technology to the gate-level standard cell and peripheral circuit modules and then to the one sub-array (or a macro as defined in this article). Then multiple sub-arrays will form one processing element (PE), and multiple PEs will form one tile with H-tree–based interconnect routing. An arbitrary neural network model could be mapped with a number of tiles.

### New Features of NeuroSim

Compared with the last version of NeuroSim (Chen et al., 2018), many new modules and features are added in this version.

**FIGURE 2:**
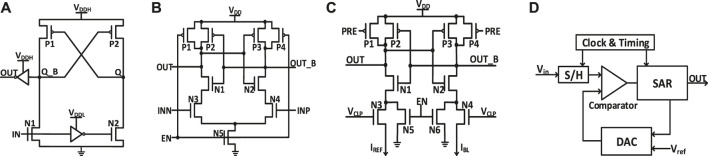
Schematics of **(A)** level shifter; **(B)** voltage sense amplifier (VSA); **(C)** current sense amplifier (CSA); **(D)** successive approximation register (SAR) ADC.

**FIGURE 3:**
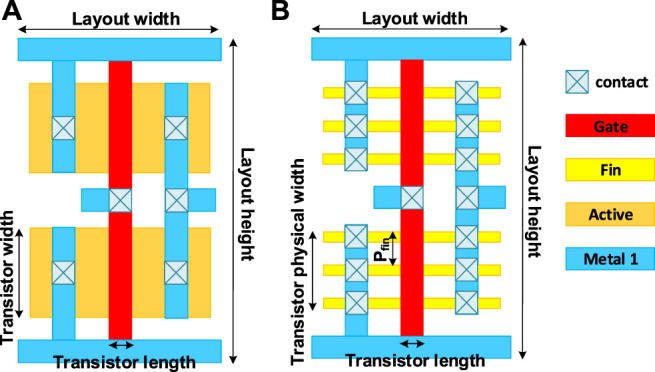
Layout of inverter cells for **(A)** bulk and **(B)** FinFET.

**FIGURE 4:**
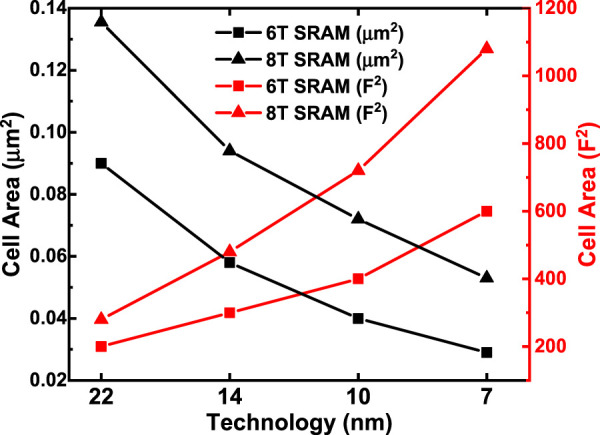
Scaling trend of SRAM cell area with technology nodes (assuming F is the same as the technology node).

• Level-shifter is normally required for RRAM (or PCM/FeFET) array to support the need of higher write voltage (than logic V_DD_). Now, a level-shifter is added as a peripheral module and will be validated later.
• Different types of ADCs are supported such as Flash ADCs using voltage-mode sense amplifiers or current-mode sense amplifiers and successive approximation register (SAR) ADC, as shown in Figure 2. They have trade-offs in the area/power and latency. For each technology node, latency and energy data from Cadence simulation are collected with sweeping of a reasonable dynamic voltage (or current) range and then are fitted with polynomial functions for fast estimation of NeuroSim, given real traces from the workloads.
• Inverter, NAND, and NOR gates based on FinFET technologies (down to 7 nm) are optimized considering the layout rule. Figure 3 shows the FinFET-based inverter gate layout. It should be pointed out that FinFET decouples the physical width (determined by the Fin pitch) and the electrical width (determined by the Fin height).
• The technology file is updated for FinFET. The default transistor models in NeuroSim were calibrated with the PTM model (PTM, 2011), which is available to the public and has a wide range of technology nodes from 130 to 7 nm. However, as the PTM model (of 14, 10 and 7 nm) was proposed far earlier than the industry adoption of FinFET, their prediction of Fin geometry actually deviated from the actual values today. We corrected the Fin height, width, and pitch following the recent trends in leading foundries and made some corresponding changes in the standard cell height/width and interconnect wire pitch, and switched to the assumption of using a maximum electrical width/or fin number in the standard cell for digital circuit design. The detailed values are shown in Table 1.
• A Scaling trend of the SRAM cell area with technology nodes is calibrated and shown in Figure 4. Since the technology node name F deviates from the transistor physical dimensions in the recent generations, the SRAM cell area that is normalized to F^2^ significantly increases in 14 nm and beyond.
• The H-tree–based routing between memory arrays is optimized with a low-swing interconnect to improve energy efficiency.
• The extra-large SRAM buffers are split into smaller block buffers for a more realistic and efficient performance estimation.
• The peripheral mux used to be sized up significantly to avoid large voltage drop for a memory device with small on-state resistance (Ron). Considering the DNN model sparsity, the sizing of mux is decided by the average column resistance, instead of the worst-case all “on” resistance to alleviate the area overhead.
• Latency is measured by clock cycles, instead of directly accumulating the critical path of each module. The clock period is decided by the sensing cycle, which is the critical path from giving input to the memory array till the ADC generating the digital partial sum as this is an analog process and no digital buffer could be added in between. The latency of other digital modules is measured cycles needed for the processing because their timing could be adjusted by adding digital buffer.

**TABLE 1:** Updated transistor model of bulk (130–22 nm) and FinFET (14–7 nm) technologies.

Technology (nm)	Bulk						FinFET		
	130	90	65	45	32	22	14	10	7
Fin pitch (nm)							48	36	30
Fin height (nm)							37	42	52
Fin width (nm)							8	6	6
NMOS width of bulk (nm) / #Fin of FinFET	907	689	507	352	267	198	3	3	2
PMOS width of bulk (nm) / #Fin of FinFET	1,809	1,191	850	587	401	262	3	3	2
Gate length (nm)	75	55	35	30	28	26	22	20	18
Standard cell layout width (nm)	988	684	494	342	243	167	143	104	78
Standard cell layout height (nm)	3,640	2,520	1,820	1,260	896	616	462	336	250

### Transistor and Peripheral Circuit Modules

**FIGURE 5:**
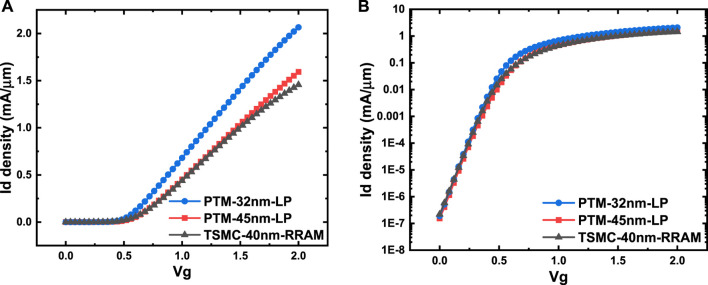
I_d_-V_g_ comparison of PTM model and TSMC PDK in **(A)** linear-scale and **(B)** semilogarithmic scale.

The default transistor models in NeuroSim are calibrated with a predictive technology model (PTM) (PTM, 2011), which is available to the public and has a wide range of technology nodes from 130 to 7 nm. However, it is known that the commercial foundry process may differ noticeably from the PTM model. Figure 5 shows the comparison of the I_d_-V_g_ curve between the PTM model and TSMC PDK. In this validation, the transistor parameters are directly extracted from TSMC 40-nm RRAM PDK and specifically set in the NeuroSim transistor library, including device W/L, the supply voltage (V_DD_), threshold voltage (V_TH_), gate and parasitic capacitance, and NMOS/PMOS on/off current density. Based on these parameters, the area and intrinsic RC/power model of standard logic gates can be calculated analytically using the formula, as discussed in Ref. Chen et al. (2018); thus, the performance metrics of each sub-circuit can be estimated. The transistor W/L in ADC, mux, switch matrix, and drivers are predefined according to the required drivability, while transistor W/L in the other logic gates used fixed size (to be corrected later with validation). The capacitances at the logic gate level are also fixed with their transistors’ sizing known, τ = RC and CV_DD_^2^ are calculated to estimate the module delay and dynamic energy consumption. Leakage power is also considered for sub-circuit modules and SRAM cells.

### CIM Macro Configurations

**FIGURE 6:**
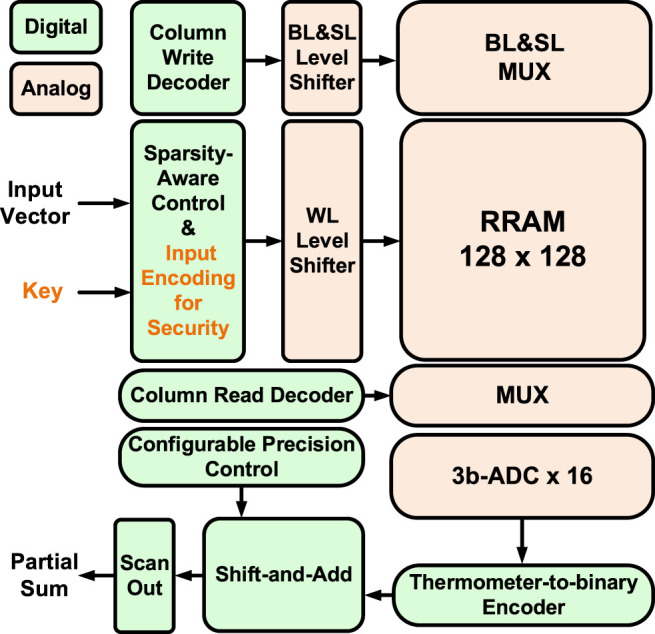
RRAM CIM macro organization that supports input zero-skip and reconfigurable weight precision. ©2021 IEEE. Reprinted, with permission, from Li et al., 2021.

**FIGURE 7:**
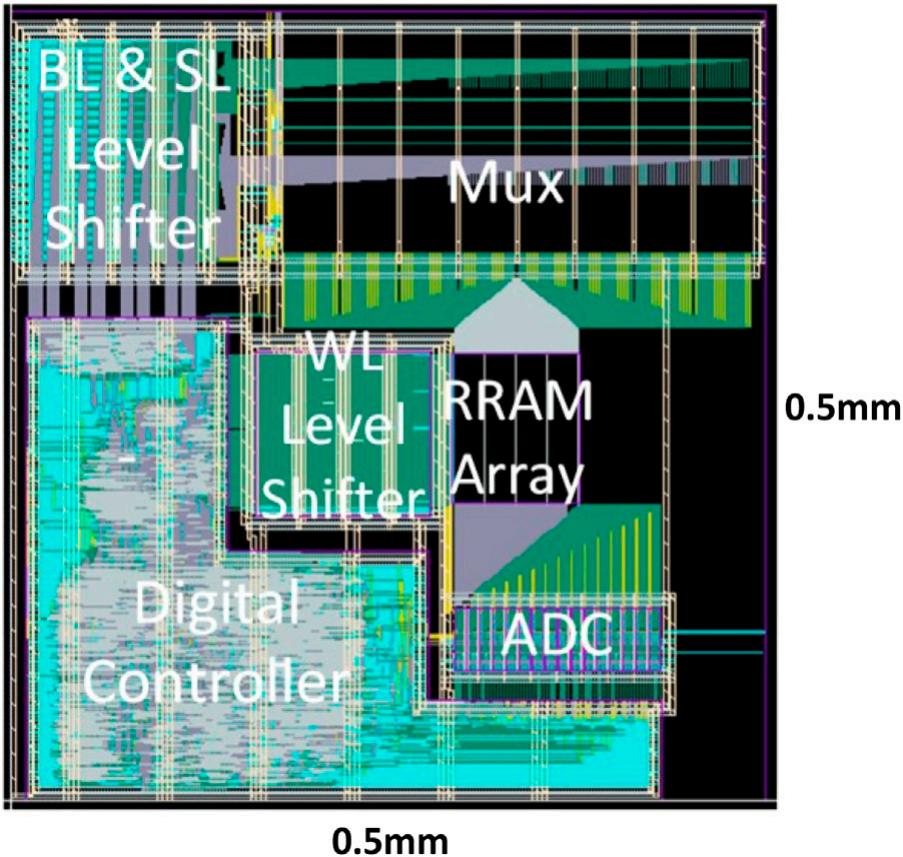
CIM macro layout implemented with TSMC 40 nm RRAM process.

In this particular design (Li et al., 2021) with TSMC 40-nm RRAM, the CIM macro could support MAC operation with zero-skip and reconfigurable precision for DNN inference. The input sparsity-aware controller counts the number of 1’s in the input vector, and the scanned rows are asserted in parallel once the counter reaches the threshold (7 in this design, considering the ADC sensing range and the practical RRAM on/off ratio). By skipping the 0’s in the input, only meaningful ADC conversions take place to improve throughput and energy efficiency. Flexible weight precision (1/2/4/8 bits) is supported to suit the optimized quantization levels for a variety of DNN models. On-chip shift-add and accumulator adaptively justify the different significances of weight bits and accumulate the partials sums in the digital domain. Each 3-bit ADC consists of seven voltage-mode sense amplifiers (VSAs) and is shared among eight columns as the RRAM cell pitch is much smaller than the size of the ADC. One reference voltage (Vref) is required for each VSA. For the ease of routing, the data column and the reference column are interleaved in a 256 × 256 physical array, but the actual computation array size is 128 × 128. Overall, the simulator settings are kept consistent with the actual macro and are summarized in Table 2. Figures 6, 7 separately show the macro organization and physical layout.

**TABLE 2:** Table of simulator settings.

Technology	TSMC 40 nm w/RRAM
Array size	256 × 256 (only 128 × 128 in computation)
ADC precision	3-bit
Weight precision	1/2/4/8 bit
Operating voltage	0.9 V
Rows turned on simultaneously	7

## NeuroSim Validation

### Analog Modules: RRAM Array, Level Shifter, Mux, and ADC

In the validation of analog sub-circuits, we mainly care about the RRAM array, level shifter, mux, and ADC. We will compare the area, latency, and energy consumption between NeuroSim simulation and the actual macro, as shown in Table 3.

**TABLE 3:** Analog module validation.

Module	Area (um^2^)		Latency (ns)		Energy (pJ)	
	NeuroSim	Real chip	NeuroSim	Real chip	NeuroSim	Real chip
Level shifter	256 × 28xαx3 = 30,966	12,084 (WL)+19,505 (BL+SL) = 31,589	1.12		1.02	0.99
Mux	459	5,400	0.06		0.07	
ADC	4,271		5		74.50	
Array	256 × 256 × 0.12 = 7,864	7,864	0.19		4.99	
(Mux decoder)	Counted in control part of digital modules		0.37		Counted in control part of digital modules	
(ADC encoder)			0.02			
Total	43,560	44,853	6.76xβ	∼10	80.58	86.79

**FIGURE 8:**
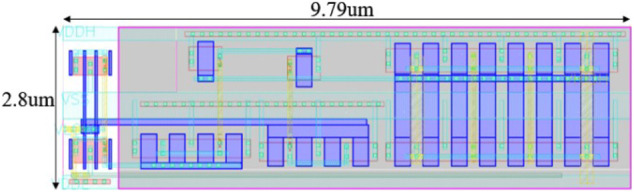
Layout of level shifter.

Area: First, the RRAM cell size is a user-defined parameter in terms of F^2^ (75 F^2^ in this design) to estimate the array area according to the array size. In the simulator, the gate area is estimated according to transistor W/L and pitch requirements in the layout rules. In general, logic transistors with minimal length are utilized to constitute the sub-circuit modules. To simulate the I/O transistors in the level shifter, the gate layout width is multiplied by 2.5 times considering the poly width and the gap between gate polys in the PDK; the gate layout height is also multiplied 2.5 times to simulate the practical gate area measured in the macro design. After these corrections, the simulator shows 2.8 um × 10 um per level shifter unit, which is quite close to the measurement on the actual layout (Figure 8). There are totally 256 × 3 level shifters for WL, BL, and SL for the entire array size of 256 × 256. By comparison with the actual area measured in the layout, a wiring area factor α = 1.44 will be imposed on the level shifter for calibration. ADCs and their mux are located together on the layout occupying about 5,400 um^2^ (the ADC block labeled in Figure 7), and the simulator estimates a result of 4,730 um^2^ with acceptable error by its default settings. The other visible mux block labeled in Figure 7 is for selecting the signal to BL and SL for programming the memory cells.

Latency: The chip could operate around 200 MHz with the digital blocks only, but the clock frequency drops to 100 MHz (post–layout simulation, 110 MHz for pre-layout) when the analog modules are included. It means the critical path is within the analog modules and it is the sensing delay from activating the level shifters to the currents summing along the columns till the ADCs converting the digital outputs. The sensing delay in the actual macro is ∼10 ns. The latency of each module estimated by NeuroSim is listed in Table 3. A latency factor β = 1.4 will be utilized in the simulator based on the comparison.

Energy: The energy consumption of analog modules is measured by SPICE simulation. In NeuroSim, the energy estimation of ADCs is also based on a lookup table–like fitting function with various weight patterns and Vref swept that are predefined by SPICE simulations. Other digital-like modules utilize CV^2^ as the dynamic energy estimation. Leakage power is also considered in NeuroSim, but the values are typically small. With precise settings demonstrated in *NeuroSim Settings* section, the estimation of NeuroSim is sufficiently accurate, as shown in Table 3.

### Digital Modules: Shift-Add, Accumulator, and Controller

The breakdown performance of digital sub-circuits in the macro design is not easy to extract because they are together automatically synthesized through register transfer-level (RTL) codes. For simplicity, we consider digital modules as only three classes to be validated: shift-add, accumulator, and control circuits. The sparsity-aware controller, encoder, and decoders are all categorized as control circuits. It is noted that although zero-skipped input is supported in this macro to improve throughput and energy efficiency, our pre-layout SPICE simulation and NeuroSim estimation are both based on 0% input sparsity (no zero-skip).

Area: From the actual macro’s digital design, the number of different types of gates and their corresponding areas can be extracted to validate the prediction. In order to support reconfigurable weight precision, the D-type flip-flops (DFFs) in the shift-add and accumulator are required to accommodate the largest precision (8-bit), and the adders have to be prepared for each precision (1/2/4/8-bit). We confirmed that the settings in NeuroSim could support the function and are similar as those in the actual chip, as shown in Table 4. In NeuroSim, the DFF contains four transmission gates, four inverters, and another four inverters for clock; the adder consists of nine NAND gates per bit. Although the exact number and types of gates cannot be guaranteed to be the same as the actual chip, the area comparison shown in Table 5 is already close to the default models in NeuroSim. Unlike shift-add and accumulator, control circuits might consist of all types of gates and the composition can be quite diverse in different designs. Therefore, all the gates in the controller are normalized to the inverter gate count according to their area for simulation simplicity. The inverter layout height in NeuroSim is multiplied by 1.84 to mimic the actual inverter area on this PDK. After the calibration, Table 5 shows that the overall area estimation of digital modules is quite accurate.

**TABLE 4:** Shift-add and accumulator settings for reconfigurable precision.

Module		1-bit weight	2-bit weight	4-bit weight	8-bit weight	NeuroSim	Real chip
Shift-add	#DFF		64 registers × 5 bit/register	32 registers × 7 bit/register	16 registers × 11 bit/register	16 registers × 11 bit/register = 176	176 DFFs
	#Adder bit		16 adders × 3 bit/adder	16 adders × 3 bit/adder	16 adders × 3 bit/adder	16 adders × 3 bit/adder × 3 = 144	144 full adders +16 half adders
Accum	#DFF	128 registers × 8 bit/register	64 registers × 10 bit/register	32 registers × 12 bit/register	16 registers × 16 bit/register	128 registers × 8 bit/register = 1,024	1,216 DFFs
	#Adder bit	16 adders × 7 bit/adder	16 adders × 9 bit/adder	16 adders × 11 bit/adder	16 adders × 15 bit/adder	16 adders × (7 + 9+11 + 15) bit/adder = 672	704 full adders +112 half adders

**TABLE 5:** Digital modules validation.

Module		Area (um^2^)		Latency	Energy (pJ)	
		NeuroSim	Real chip		NeuroSim	Real chip
Shift-add	DFF	719	681	1 cycle = 10 ns	2.504 x γ = 1.25	1.25
	Adder	662	663		0.81 x δ = 0.12	0.15
	Inverter	2,133 INV = 1,336	1,334		3.31 x ϵ = 0.17	0.33
Accumulator	DFF	4,968	4,706	1 cycle = 10 ns	17.3 x γ = 8.65	8.37
	Adder	3,089	3,291		3.99 x δ = 0.60	0.60
	Inverter	10,869 INV = 6,808	6,797		16.88 x ϵ = 0.84	0.80
Control	DFF		7,334		26.96 x ζ = 2.97	3.01
	Inverter	10,485 INV = 6,569	6,558		16.28 x ϵ = 0.81	0.75
Total		31,893	31,386		15.41	15.26

Latency: As we pointed out earlier, the sensing cycle of the RRAM array is typically the critical path of the entire chip as the digital blocks can always be partitioned into multiple stages to be hidden within this analog critical path delay. Therefore, we propose counting the number of operations for digital modules. Each operation of shift-add or accumulator is one cycle according to the timing. For the entire DNN processing, we estimated the chip-level latency as the total number of clock cycles to complete the computation in a layer-by-layer manner.

Energy: Table 5 shows the comparison of dynamic energy consumption of digital modules. The energy of actual chip is extracted from SPICE simulations. As most gates actually do not switch during run-time, switching activity factors should be considered in real workloads. As the DFFs are able to accommodate the largest precision, most DFFs are on operation when the real chip is tested under 8 bits. While the adders are prepared for each precision, most of gates are inactive in practice. Therefore, we set activity factors γ = 50% and δ = 15% separately for DFF and adder of shift-add and accumulator. The normalized inverters and DFFs to simulate the control circuits are employed with factor ϵ = 5% and ζ = 11%.

### Post-Layout Calibration

The above performance comparison (except sensing delay) is based on pre-layout SPICE simulation. For chip-level energy efficiency, the actual macro could run at 10 TOPS/W with 0% input and 50% weight sparsity, where we can derive that it costs 3,151 pJ to compute the entire array (128 × 128 × 2 operations). As a comparison, NeuroSim predicts 3,178 pJ after the calibration. In order to reflect the silicon data, the post-layout performance drop is also considered in our validation, as shown in Table 6. In post-layout SPICE simulation, the macro has an energy efficiency of 8.48 TOPS/W with the same input and weight patterns, which derives that 3,864 pJ is required to compute the entire array. Therefore, a factor η = 1.22 is imposed to estimate the chip-level post-layout dynamic energy consumption.

**TABLE 6:** Chip-level pre- and post-layout energy comparison.

Energy for whole array	NeuroSim	Real chip
Pre-layout	3,177.75 pJ	10.4TOPS/W → 3,150.8 pJ
Post-layout	3,177.75 pJ×η	8.48TOPS/W → 3,864.2 pJ

## Benchmark

In this section, we evaluate the impact of the aforementioned calibration factors on the DNN+NeuroSim framework by implementing the VGG-8 model on CIFAR-10 dataset, testing on various technologies and memory devices with a general architecture and operation mode, following the methodologies reported in Ref. Peng et al. (2019). The simulation is set up across versatile device technologies (HfOx RRAM (He et al., 2020), TaOx/HfOx RRAM (Wu et al., 2018), PCM (Kim et al., 2019), and FeFET (Ni et al., 2018), as shown in Table 7. SRAM-based CIM accelerators are evaluated at both 22 and 7 nm, and eNVM-based ones are evaluated at 22 nm as 22 nm is the state-of-the-art node where the eNVMs are integrated. Considering the read-noise and on/off ratio, the 4-bit/cell is assumed for eNVMs, except the 2-bit RRAM from Winbond (He et al., 2020). The subarray size is 128 × 128. A 4-bit precision ADC is utilized for 1-bit SRAM cells, with an inference accuracy of 92%; while a 5-bit precision ADC is utilized for multi-bit eNVMs to maintain an inference accuracy of 91% (Peng et al., 2019). Relatively high precision with 8-bit weight and 8- bit activation is also used to ensure no accuracy loss. A full 128-row parallel operation is assumed for the most efficient calculation. The number of operations is normalized to 8-bit, regardless of the memory cell precision.

**TABLE 7:** Benchmark results of CIM accelerators on VGG-8 for CIFAR-10 and ResNet-18 for ImageNet, based on SRAM (at 7 and 22 nm), and reported eNVM devices (assumed at 22 nm).

Technology node (LP)	7 nm	22 nm				
Device	8T-SRAM	8T-SRAM	RRAM [12]	RRAM [13]	PCM [14]	FeFET [15]
MLSA-ADC precision	4-bit	4-bit	5-bit	5-bit	5-bit	5-bit
Memory cell precision	1-bit	1-bit	2-bit	4-bit	4-bit	4-bit
Ron (Ω)	/		6 k	100 k	40 k	240 k
On/off ratio	/		150	10	12.5	100
VGG-8 (8-bit activation; 8-bit weight) on CIFAR10, with novel weight mapping and dataflow						
Area (mm^2^)	13.34	61.92	45.55	25.57	25.57	25.52
Memory utilization (%)	98.73%	98.73%	96.86%	93.47%	93.47%	93.47%
Clock period (ns)	2.98	4.87	2.05	2.02	2.22	2.30
L-by-L latency (ms)	2.09	3.60	1.46	1.30	1.43	1.48
L-by-L dynamic energy (uJ)	31.27	58.69	37.75	16.73	17.32	16.07
L-by-L leakage power (mW)	2.71	1.73	0.63	0.33	0.33	0.33
Compute efficiency (TOPS/mm^2^)	0.044	0.006	0.019	0.037	0.034	0.033
Pre-layout energy efficiency (TOPS/W)	31.87	18.48	31.63	71.25	68.69	73.72
Post-layout energy efficiency (TOPS/W)	26.12	15.14	25.93	58.40	56.11	60.43
Before calibration						
Area (mm^2^)	13.34	60.25	31.18	17.88	17.88	17.64
Compute efficiency (TOPS/mm^2^)	0.147	0.027	0.057	0.118	0.118	0.120
Energy efficiency (TOPS/W)	47.66	21.78	40.89	85.44	82.12	89.14
ResNet-18 (8-bit activation; 8-bit weight) on ImageNet, with novel weight mapping and dataflow						
Area (mm^2^)	16.77	80.37	62.04	39.68	39.68	39.61
Memory utilization (%)	94.59%	94.59%	91.42%	86.64%	86.64%	86.64%
Clock period (ns)	2.98	4.87	2.05	2.02	2.22	2.30
L-by-L latency (ms)	22.75	39.14	13.39	11.53	12.67	13.12
L-by-L dynamic energy (uJ)	148.50	275.81	197.03	92.62	96.00	89.18
L-by-L leakage power (mW)	3.29	2.11	0.80	0.50	0.50	0.50
Compute efficiency (TOPS/mm^2^)	0.014	0.002	0.007	0.012	0.011	0.011
Pre-layout energy efficiency (TOPS/W)	25.87	15.93	26.68	56.41	54.27	58.07
Post-layout energy efficiency (TOPS/W)	21.20	13.06	21.87	46.24	44.48	47.60

**FIGURE 9:**
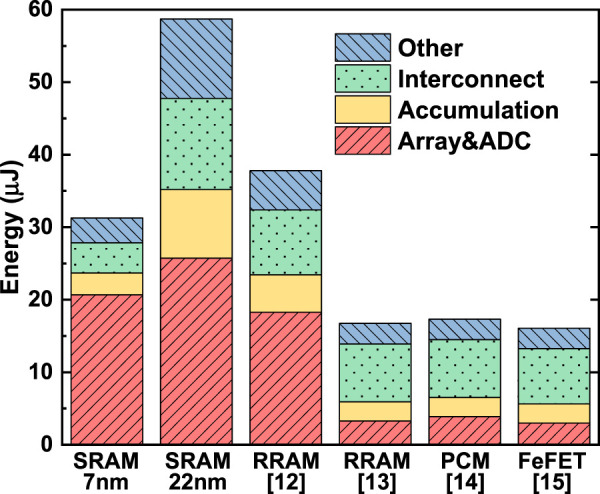
Energy breakdown of CIM accelerators on VGG-8 for CIFAR-10, based on SRAM (at 7 and 22 nm), and reported eNVM devices (assumed at 22 nm).

The general conclusions stay the same as Ref. Peng et al. (2019). First, at the same technology node, eNVM-based designs outperformed the SRAM-based designs in both energy efficiency (in the unit of TOPS/W) and compute efficiency (in the unit of TOPS/mm^2^). Second, devices with higher on-state resistance (Ron) such as FeFET show substantial improvements in energy efficiency. Third, SRAM at the leading-edge node (e.g., 7 nm or beyond) still show competitive energy efficiency and outstanding compute efficiency. Compared to the previous results before the validation, the new benchmark results show that the areas of eNVM-based designs are increased substantially owing to the calibration for the level-shifter area. The compute efficiency in all the design significantly decreases mainly because of the adopted clock cycle–based method to measure the latency. The pre-layout energy efficiency is reduced mainly as a result of larger transistor size utilized after update, while the calibration on energy consumption of DFFs and adders somehow offset the more leakage caused by longer latency and the longer interconnect distance caused by the larger area. The post-layout energy efficiency is further dropped as a direct result of the calibration. The energy breakdown of simulated accelerators on VGG-8 for CIFAR-10 is shown in Figure 9. The devices with high Ron cost much less energy on the memory array charging and ADCs; devices with high cell precision could effectively reduce the operation of bit shift-and-add, thus reducing the energy consumption on accumulation; a smaller chip area contributes to less interconnection energy.

In this work, we also explore the scalability of the framework toward larger networks for more complex problem. The benchmark results of the ResNet-18 model on ImageNet dataset are also shown in Table 7, where the trend is similar as VGG-8 on CIFAR-10. The inference under 8-bit weight and 8-bit activation could reach 69% top-1 accuracy of ImageNet. The overall chip area increases by 25–50%, compute efficiency decreases by ∼70%, and energy efficiency decreases by ∼20% for ImageNet compared to CIFAR-10 workloads. In this version of the released framework, we assume a custom chip design for specific DNN models where all the weights are stored on chip. For the designs with chip area constraints where the weight reloading from off-chip DRAM is unavoidable, the readers could refer to the relevant discussions in Lu et al. (2020). For the reconfigurable chip design where one chip instance is able to support various DNN models, the readers could refer to the relevant discussions in Lu et al. (2021).

## Discussion

The related works in this field include the following reported simulators. NVSim (Dong et al., 2012) is a memory-oriented simulator, and its peripheral circuit modules do not support CIM functions. Other reported CIM-oriented simulator platforms such as MNSIM (Xia et al., 2018) and TxSim (Roy et al., 2021) have demonstrated powerfulness in the design space exploration or the device nonideality analysis, but they may have limited considerations either on the algorithm accuracy or on the hardware performance metrics. RxNN (Jain et al., 2020) is capable of various device and circuit nonideality analyses and rough energy estimation. Compared with RxNN, our work makes more comprehensive considerations on the hardware performance estimation. An IBM Analog AI HW Kit (IBM, in press) and CrossSim (CrossSim, 2018) only focus on the neural network accuracy estimation without the hardware performance estimation. PIMSim (Xu et al., 2018) is an architectural simulator for process in memory (most for near DRAM processing) with compatibility for traditional computer architecture simulator GEM5.

The prediction of NeuroSim is validated against the post-layout simulation of an actual 40 nm RRAM-based CIM macro design. Some adjustment factors are introduced: α = 1.44 for the wire areas in the level shifter; β = 1.4 for the sensing cycle as the critical path; γ = 50% and δ = 15% separately for dynamic energy of DFFs and adders in shift-add or accumulators; ϵ = 5% and ζ = 11% for dynamic energy of inverters and DFFs in control circuits; and η = 1.22 for a post-layout energy increase. After these calibrations, the chip-level simulation from NeuroSim is quite accurate with error under 1%.

However, we admit some inevitable limitations of this validation. First, the factors might be overfitted for this specific design. Limited by the available resources, it is unrealistic for us to have more chips fabricated with different technologies or design options. Although there are some other reported CIM macros developed by other groups, the lack of detailed design information and performance breakdown prevent using them for such validation. Second, even with our own CIM macro, the performance breakdown is not precise enough. For example, in NeuroSim, the latency is considered as the accumulation of the critical path delay of each module, while for the real chip, we could only get an overall estimation according to the clock cycle. Third, the calibration mainly focuses on the sub-array level as there is no large-scale multi-macro system with eNVM-based CIM accelerators as of today. Some additional factors may be required to capture the system-level activity rate of accumulators and buffer access frequency. Nevertheless, we believe this calibration with actual silicon implementation could offer an important reference and make the estimation of NeuroSim more convincing and reliable for the growing community of this simulator.

## Data Availability

The datasets presented in this study can be found in online repositories. The names of the repository/repositories and accession number(s) can be found below: https://github.com/neurosim. Open-source code availability: NeuroSim source code used in this work is publicly available at https://github.com/neurosim/DNN_NeuroSim_V1.3.
